# Determinants influencing antibiotic use in Singapore's small-scale aquaculture sectors: A qualitative study

**DOI:** 10.1371/journal.pone.0228701

**Published:** 2020-02-25

**Authors:** Jane Mingjie Lim, Minh Cam Duong, Li Yang Hsu, Clarence C. Tam

**Affiliations:** 1 Saw Swee Hock School of Public Health, National University of Singapore and National University Health System (NUHS), Singapore, Singapore; 2 London School of Hygiene & Tropical Medicine, London, England, United Kingdom; University of Lincoln, UNITED KINGDOM

## Abstract

Singapore’s Antimicrobial Resistance (AMR) national strategic action plan includes inappropriate use of antimicrobials in food-producing animals as a specific priority. Although the use of antibiotics and other drugs are monitored by regulatory bodies, food fish farmers are allowed to buy and administer antimicrobials without a veterinary prescription. We conducted a qualitative study of Singaporean food fish farmers to understand patterns and determinants of antibiotic use, their knowledge of antibiotic resistance, as well as perceptions of on-farm infection prevention and control measures. During the interview, participants were asked about their farming processes, farm infrastructure, antibiotic use and any disease prevention measures. Thematic analysis of participants’ interviews showed that antibiotic for growth promotion and infection prevention was uncommon among local food fish farmers. The following three main themes influenced participants’ decisions to use antibiotics in their practice: 1) individual factors, 2) local regulatory factors as well as 3) market-related factors. Individual factors included their personal experience and knowledge both with antibiotics as well as with alternate options. In terms of local regulatory factors, we found that regular oversight was a strong deterrent in antibiotic use. Last, at the market level, the relatively high price of antibiotics in Singapore coupled with stiff competition was a strong disincentive for participants to use antibiotics in their farming practice. These factors were also influential in their relationships with local regulatory bodies as well as their counterparts. Although industries differ significantly across countries, lessons learnt from Singapore’s food fish farming demonstrate the importance of an environment where multi-dimensional factors come together to discourage the irrational use of antibiotics in food animal production. In addition, our results allow greater insight into food fish farmers’ perspectives on infection control and form a basis from which further research work can be undertaken.

## 2. Background

The aquaculture sector plays a crucial role in global food security. Worldwide per capita fish consumption has increased dramatically in the past decades and has surpassed meat consumption from all terrestrial animals. According to the Food and Agriculture Organization of the United Nations [1], 88% of total global fish production in 2016 was for direct human consumption (171 million tonnes), with worldwide projected consumption expected to reach 201 million tonnes in 2030. With Asia as the major contributor to this expansion, intensification of the continent’s aquaculture sector has been accompanied by frequent outbreaks of infectious diseases, increasing the use of both prophylactic and therapeutic treatments such as antibiotics and probiotics in farming practices [2].

Limited data are available on the type and amounts of antibiotic use in aquaculture, especially in developing countries where the majority of aquaculture production occurs [3]. Additionally, in these settings, farmers who administer the antibiotics often lack awareness about the appropriate use of these drugs. For instance, a study conducted by Holmström et al [4] showed that a large proportion of shrimp farmers in Thailand used antibiotics prophylactically—either in the feed or through baths—some on a daily basis. Similarly, another cross-sectional study conducted on freshwater aquaculture in Vietnam [5] indicated a general lack of knowledge about the purpose and proper usage of antibiotics by aquaculture farmers. Extensive use of antibiotics in aquaculture can result in the development of reservoirs of antimicrobial-resistant bacteria in fish, aquatic animals as well as the aquatic environment, increasing potential adverse consequences for both humans and animals [6, 7].

To mitigate human health risks from antimicrobial use in food producing animals, a tripartite agreement has been formed across the Food and Agriculture Organization of the United Nations (FAO), the World Organization for Animal Health (OIE) and the World Health Organization (WHO) to monitor health risks in human and animal health, plant production and the environment, and to enable better implementation of the Global Action Plan on Antimicrobial Resistance [8]. In addition, WHO-FAO-OIE recommendations have emphasised the urgency of reducing unnecessary antibiotic use in agriculture, particularly the phasing out of medically important antimicrobials for animal growth promotion. Following these recommendations, the European parliament (P8_TA(2018)0429) [9] has approved legislation to ban the use of human antibiotics in veterinary medicine and the use of animal antimicrobials for prophylaxis and metaprophylaxis without prescription.

In line with World Health Assembly’s endorsement of an AMR global action plan, Singapore launched its national strategic action plan to address AMR in November 2017 [10]. The action plan includes as a specific priority to reduce inappropriate use of antimicrobials in food-producing animals. The Agri-Food and Veterinary Authority of Singapore (AVA) already has in place licensing conditions for the manufacture of animal feed, and has implemented guidelines for antimicrobial use in all food-producing animals to ensure that no antimicrobials are used for growth promotion. Current directives also state that farmers who use antibiotics need to observe a minimum withdrawal period before the animals or animal products can be slaughtered or sold to prevent antibiotic residues in food. Further, the action plan aims to promote better animal management practices as well as the use of vaccines in livestock, pets and fish to reduce the overall reliance on antimicrobials.

Although Singapore has a small agriculture sector, the local food fish industry is thriving, with both coastal and freshwater farms that produce mainly grouper, seabass, snapper and tilapia. There are currently 125 fish farms in Singapore, producing a total of 4,851 tonnes of fish in 2016, accounting for about 10% of total fish consumption locally. At time of study (2017), food fish farmers are allowed to buy and administer antimicrobials without a veterinary prescription in Singapore. AVA then monitors the use of antibiotics and other drugs through veterinary drug wholesalers’ records of sales to farms and vet clinics. However, little is known about patterns and determinants of antimicrobial use in Singapore's food fish farming sector. Hence, we conducted a qualitative study of Singaporean food fish farmers to better understand their knowledge of antibiotic use and antibiotic resistance, as well as perceptions of on-farm infection prevention and control measures.

## 3. Methods

### 3.1 Participant recruitment

We approached fish farmers at a workshop held by the Agri-Food and Veterinary Authority (AVA), Singapore, where we explained the study and requested permission to contact them at a later date to arrange an in-depth interview. We identified additional farmers through websites of Singapore fish farms. Potential participants were contacted by telephone by a trained researcher who explained the purpose of the study and invited farmers to take part. The researcher arranged an interview with willing participants on a convenient date. Participants were asked to choose a convenient location for the interview. Fifteen interviews were conducted over a period of eight months from October 2017 to June 2018.

### 3.2 In-depth interviews

Farmers who were willing to participate were interviewed face-to face by two trained researchers (CD, JL) and all interviews were audio-recorded. Participants gave written informed consent, as well as permission for the interview to be recorded. If a participant refused to be audio recorded, detailed notes about the interview were taken instead. For Mandarin-speaking participants in this study, both informed consent and the corresponding interviews were conducted in Mandarin. No personal identifiers were audio recorded.

The interviews followed a semi-structured approach and the same interview guide was used for all interviewees. The interview guide was developed through reading previous literature as well as discussion with AVA veterinarians. Key topics were then identified and incorporated into the interview guide. To ensure that the questions would be well understood by participants, the interview guide was first piloted with two fish farmers. Minor changes were made to the guide at this stage after discussion with study team members. During the interview, participants were first asked about themselves and their farm, such as how long they had been farming, the size of their farm, species of fish on their farm as well as their farm processes and infrastructure. They were then asked to describe their antibiotic use as well as other farm practices, including any disease prevention measures. Where necessary, probing questions were asked to elicit further information on topics related to the scope of the project.

### 3.3 Data analysis

Interviews ranged between 25 to 120 minutes each, and all audio recordings of the interviews were transcribed verbatim. A random sample of interview transcripts was checked by the researchers who interviewed the farmers (CD, JL) and compared with the recordings to ensure accuracy of transcriptions. Transcriptions were then stripped of any identifiers and imported into NVivo Version 10 (QSR International, London, UK) to facilitate data coding, retrieval and analysis.

Thematic analysis using an inductive (data driven) approach was used to analyse the data. Initially, two researchers (CD, JL) independently coded a subset of five interviews and each developed a preliminary set of codes for analysis. The two independent code sets were then compared and reviewed by both researchers to develop a consolidated codebook for the rest of the analysis. Minor discrepancies during this process were resolved through discussion. Final modifications were then made to improve the comprehensibility of the framework. Using the final coding framework, JL and CD then coded two transcripts collaboratively to ensure agreement. JL then coded the remaining transcripts independently. The content of each code was available to all authors for subsequent validity checks. For the purposes of this analysis, we coded according to the key topics described below. Participant quotations are provided to illustrate our findings.

### 3.4 Ethical consideration

Ethical approval was obtained from the National University of Singapore Institutional Review Board (reference code: S-17-323). In the results section below, direct quotations are used to illustrate the emerging themes in the data. Individual participants are depicted by a study identifier at the end of each quote to maintain participant anonymity.

## 4. Results

### 4.1 Background (description of participants/general antibiotic use)

The 15 farmers included in our study were all male and had between two and 37 years of experience in the food fish farming industry, with a median of 12 years. More information on participant and farm characteristics can be found in Table 1.

**Table 1:** 

	Number of participants n = 15 (%)
**Participant characteristics**	
Full-time farmer	14 (93.3%)
Part-time farmer	1 (6.7%)
**Farming system**	
Traditional cage farming	15 (100%)
**Farm setting**	
Coastal farm	13 (86.7%)
Land-based farm	2 (13.3%)
Farm size*	
Half hectare (5,000 square meters)	10 (66.7%)
1 hectare (10,000 square meters)	4 (26.7%)
4 hectares (40,000 square meters)	1 (6.6%)
Production**	
>17 tonnes (17,000 kilograms)	15 (100%)
Number of species per farm***	
1–2 species	9 (60%)
3–4 species	5 (33.3%)
>4 species	1 (6.7%)

* At time of study, the Agri-Food and Veterinary Authority of Singapore (AVA) rented out sea space in units of 0.5 hectares.

** For annual license renewal of sea space, AVA stipulates a minimum production level of 17 tonnes (17,000 kilograms) of fish per half-hectare of rented sea space per year. However, exceptions are granted to farms affected by disease outbreaks in the same year.

*** Fish species include grouper, milkfish, sea bass, snapper, mullet, tilapia, pomfret, trevally, threadfin, catfish, robalo (snook), and perch. Crustaceans include lobster, prawn and other barnacles.

When asked about antibiotic use, no participant said that they were using antibiotics regularly in their farming at time of the interview. A few fish farmers reported using antibiotics as a last resort when their fish remained sick after trying other treatment methods. Participants also discussed some circumstances when they would use antibiotics, including symptoms such as loss of appetite and slow swimming. For participants who were coastal fish farmers, the most common first-line treatment described for any signs of illness was freshwater treatment:

*“…because ours are sea water fish, when salt water touch fresh water*, *the fish bacteria cannot take it and it dies.” (ID007)*

If the freshwater bath did not work, several participants described the use of formalin:

“*We bathe it once*, *if still cannot*, *we will add some formalin*. *Formalin is normally AVA recommend*. *Okay so we will add some formalin then just wash like that*. *We need to wash about a week about 2 to 3 times… if there's bacteria it’ll recover faster but if you don’t wash it*, *it’ll keep attacking and attacking the fish and it will spread and it spreads quickly*.*” (ID002)*

However, all of the food fish farmers we interviewed reported strong disincentives to use antibiotics in their farming practices. Through thematic analysis, we found that the three following major themes contributed to their decision not to use antibiotics: 1) individual factors, 2) local regulatory factors and 3) market factors.

### 4.2 Individual factors

#### 4.2.1 Personal knowledge

A key disincentive to using antibiotics was farmers' understanding about what could be used as therapeutic treatments in fish farming, and more specifically, their personal knowledge of antibiotic use and resistance. For instance, some participants showed discernment in the different treatments required for bacterial, viral or parasitic attacks:

“*It depends on what is affecting them if is a virus*, *you use antibiotics is no use* … *so antibiotics is only if they come under bacteria attack or something like that*.*” (ID001)*

Three participants mentioned refraining from antibiotic use in their farming practice due to their personal understanding about the impact of antibiotics in food production and human consumption. A common response was that *“now antibiotics cannot anyhow use*, *including those taken by humans…it’s not good for human body…you cannot let the fish eat all of these*, *it’s harmful to human*.*” (ID010)*. One participant commented on having observed the reduced effectiveness of antibiotics as well as the possible toxicity of antibiotics:

*“Interviewee: I’ve noticed that when you use antibiotics*, *[it sometimes] actually it makes it worse*

*Interviewer: Oh really*? *How?*

*Interviewee*: *Okay let's say you feed the fish, when it’s this size, you give antibiotics when they’re sick, right? Next time, I can see that you one spoon is not enough, so I want more [and] in the end the body cannot take it anymore…” (ID 005)*

A fraction of these participants also alluded to some awareness about antimicrobial resistance and conscientiousness in food production, where one participant commented:

*“I don*’*t want to live with a conscience of saying that I contribute to a [public health problem]” (ID 001)*

#### 4.2.2 Personal experience with antibiotics

Previous personal experience was another disincentive that deterred participants from using antibiotics in their current farming practice. A number of participants expressed the futility of antibiotic treatments as they did not find it effective in either preserving or increasing the survival rate of their fish (ID001). In addition, a few farmers reported side effects from fish antibiotic consumption, finding impaired growth of their fish if they ate a lot of antibiotics. Since antibiotics are usually mixed into the feed for fish consumption, one participant then added that antibiotic treatment for sick fish is inevitably prophylactic antibiotic use. One participant said:

*“Because sick fish do not really eat*, *so when you put antibiotics in the food you usually end up with the healthy fish eating the antibiotics…so what’s the point?” (ID008)*

#### 4.2.3 Personal experience with alternative practices

When asked if they had found more effective ways to prevent and curb infection, participants discussed some alternative methods with which they had more success. These methods include both conventional methods as instructed by AVA or other fish farmers as well as some methods specific to individual farmers.

### 4.3 Hygiene

Most participants who were coastal fish farmers spoke about the importance of good hygiene. While they understood that most conditions at sea were generally not within their control, they did all they could to prevent additional bacterial growth on their farming apparatus:

*“Once in the sea…the nets are dirty*, *you know? And especially when the fish rests on the net at night, that’s when parasites can cling onto them very easily…so I use a high-pressure jet spray and let my nets dry out in the sun very often. Don’t let the parasites from attacking them [fish] that means keeping the nets very clean.” (ID003)*

### 4.4 Probiotics

Although most participants said that probiotics were a plausible alternative to improve fish immunity, only a few participants said that they had tried them. The participants did not mention any specific deterring factor to using probiotics, although participants generally did not observe an increase in survival rate or yield after probiotic use. One participant shared that he continues to use probiotics:

“*We just have to believe that it must be something good and then try*. *If it is no harm*, *you can do and is not that horribly expensive… It’s just your rice water and then you have milk and your sugar–we call it lactobacillus*.” *(ID001)*

### 4.5 Nanosilver

One participant in particular spoke about his successful experiences with an unconventional mode of treatment–by injecting nanosilver into sick fish. He cited the lack of side effects as an incentive for why he has included it as a main mode of treatment in his farming practice. In addition, the participant showed some awareness about issues related to antibiotic overuse in fish farming practices.

*“Yeah*, *normally for infection we just give one jab, you know? They stop the infection, immediately. They don’t have a, silver is actually a trace mineral so they don’t have a side effect. And also bacteria and viruses have no resistance against minerals. They can build up resistance against antibiotics but they cannot build up resistance against minerals because this is about positive and negative charge.” (ID004)*

### 4.6 Local regulatory factors

#### 4.6.1 AVA’s regular inspection

Although some participants understood that current local regulation did not require eliminating antibiotics from their practice, there was clear consensus from all the participants that a major deterrent in their decision whether to use antibiotics or not had to do with AVA’s regulations and its corresponding implementation to increase awareness of antibiotic use in recent years. For instance, a common point of discussion amongst almost all the participants was AVA’s regular quarterly inspection of their farms:

“*AVA will come by every two months to our farm to take the fish*, *they will come take the fish to the lab*, *so if anything happens in the lab they will find you*, *if nothing happen then it’s okay” (ID002)*

Possibly as a result of some of AVA’s newer regulations over the past decade, a minority of participants discussed some noticeable differences with antibiotic use in Singapore’s food fish farming industry. One participant said:

*“Last time [about 10 years back], people will go to the polyclinic to buy the [human] antibiotics to use for the fish…but these few years [it’s been] stricter. We cannot [simply use] antibiotics because AVA will diagnose that your fish have antibiotics and will confiscate. It is also harmful to human” (ID010)*.

#### 4.6.2 Only able to buy medicines from one wholesaler

Most of the participants spoke about the challenges of buying antibiotics within a heavily regulated environment. Although no prescriptions are currently needed to buy fish medications, participants shared that they needed to show their farm license and other relevant identification documents to the wholesaler before they are able to get any antibiotics or other fish medication. However, a few participants mentioned that going to the designated seller was generally for compliant farmers and that there are definitely fish farmers who obtain their fish medications from somewhere else, such as neighbouring country Malaysia:

“*Lim Guan Chuan is for compliant people [and] I think they could get it from Malaysia can get it from elsewhere…” (ID009)*

#### 4.6.3 Difficulty in selling fish with antibiotics due to other regulatory environments

Another reason to not use antibiotics was the potential difficulty in selling their fish to suppliers, especially to countries with strict regulations about antibiotics in food animals, such as Australia. This challenge was shared by one participant:

*“I think [using] antibiotics here is okay, but once I got a friend at there, he’s a Caucasian, he came to me and tell me he wants to buy jinmulu (sea bass) to export to Australia…but with one condition: no antibiotics…he said [Australia] is very strict…once tested for [antibiotics]*, *the whole batch cannot enter already.” (ID007)*

### 4.7 Market factors

#### 4.7.1 Profit margin

For many of the participants, a key disincentive to using antibiotic treatments in their farming practice was the inability to justify the costs of antibiotics in relation to their overall operational costs. This issue is two-pronged: all participants shared the challenges of farming in Singapore especially as they are in constant competition from neighbouring countries that have significantly lower operational, production and manpower costs. One participant stated:

*“At least meet our cost if not give us a small profit because we have competitors from Malaysia and Indonesia*. *We have always given feedback that their production cost are much lower 30–40% at least because labour cost, fuel cost, operating on the sea is the same because sea operation in terms of compare to land cost I think if you operate a land in Malaysia with big ponds and so on it’ll be very cheap, we can’t afford to have that in Singapore because of high land cost, on the sea well, the licence fee I think is not much, one for us half a hectare is 850$ a year so that is almost like almost like good” (ID002)*

Correspondingly, the other major disincentive identified by the participants was the high cost of antibiotics in Singapore. Although no antibiotic type or brand was specified by the participants, a majority of the participants said that the cost of antibiotics that they bought in the past could cost anywhere from SGD$50–200 (USD$30–150) for 500 grams (~1 lb) of antibiotics, and for farmers with larger fish stocks, this was only sufficient for one to two uses. The high cost of antibiotics is particularly a barrier for farmers who sell lower value fish such as mullet and milk fish, for whom it generally makes more economic sense to let the fish die than to treat them with antibiotics:

*“If you are talking [about my fish]*, *they cost only 20 cents, 30 cents. My medication is worth much more than the fish… so I just let them die” (ID003)*

#### 4.7.2 Consumer awareness

Participants had mixed responses about consumers today becoming more conscious and aware about issues relating to food safety. While most regarded this as an innocuous change with little impact on their business, a small number of participants discussed the challenges of changing consumer perceptions and have become wary about adding antibiotics to their fish as it may not be acceptable to some consumers:

*“Because once they realise that you will add so-called chemicals on the so-called food product*, *people don’t want” (ID012)*

### 4.8 Potential interventions

To find out what participants thought about potential interventions in line with the Singapore AMR National Action Plan, we asked farmers for their views about the feasibility of interventions such as fish vaccination and obtaining prescriptions from licensed veterinarians for fish medications. We found that the three themes also applied to any potential interventions that could reduce the need for therapeutic antibiotic use.

#### 4.8.1 Vaccination

When asked about fish vaccinations, all of the participants were hesitant about it being an effective intervention, and questioned the feasibility of carrying out vaccinations on all fish farms:

“*How can you do that to millions of fish*? *It still doesn't make sense*. *The technology is just not ready*. *There’s no way to vaccinate the fish at that scale*.*” (ID014)*

Similar to antibiotic use, another recurring concern was the inability to justify the cost in relation to the cost of the fish, especially if they were doubtful of the effectiveness of vaccinations. A few participants shared common previous experiences with fish vaccination:

*“Don’t like to inject–inject very expensive*. *It’s a matter of the cost. If I say it is double the cost…that means my survival rate has to double, otherwise, I lose money… doesn’t make sense. One jab like 10–20 cents… last time, many years ago, we also give them [fish] jabs, also the same–they still get sick and die. Usually they say the injection is for those disease that are harder to kill, but I still think it’s redundant, waste of money… things still happen…” (ID010)*

While some participants worried about general consumers’ acceptance about vaccinated fish, there were also personal beliefs about fish farming that came into play, as some participants expressed their unwillingness to interfere unnecessarily with their fish. In particular, one participant shared his unique perspective about fish vaccinations:

*“…I don’t really want to rely on somebody just putting in the vaccine and without the vaccine you cannot do the farming. I don’t believe this rule*. *Just like human, can you believe that you cannot survive without having vaccine? Can the world’s population die by without vaccine? It’s not true…humans have the ability to survive so are the fish. It’s just give them the right things for them to make their immunity. Just like, for example, the forest, do we need the humans to go and protect the forest for millions of years? Do we need to vaccine the trees and all this? They also have virus. Do we need human intervention? Our intervention is not to destroy them, not to help them. They regenerate themselves, they have ways to, you know? So they heal themselves from the soil minerals. Soil minerals got everything. Same for the fish.” (ID004)*

#### 4.8.2 Enforcing vet prescriptions

As mentioned previously, food fish farmers in Singapore are allowed to buy and administer antimicrobials without a veterinary prescription. AVA then monitors the use of antibiotics and other drugs through veterinary drug wholesalers’ records of sales to farms and vet clinics. According to some participants, although the wholesalers provide some advice, they admit that the process of using medication in food fish farming is by trial and error. When asked about AVA’s plans to implement a veterinary drug registration system and requirement for prescriptions for all veterinary antimicrobials, including those used in aquaculture, some participants shared that one of the main challenges would be enforcing this regulation:

*“I suppose…how are you going to enforce it in the first place right…so you want to make a rule you must be able to enforce… sad to say that not all humans are prepared to say you make a rule I am very obedient I come and comply hundred percent will whatever rules you make… that*’*s human nature…” (ID008)*

One participant voiced the challenges of enforcing this as he did not find that current vet advice was helpful in his farming practice:

*“Ya, now even when you have problem*, *you tell them. They can’t do much. So help means, you must help them solve the problem. Because they do not have much knowledge, so called governing authority but they do not have much knowledge because they all these fresh graduates, you know?” (ID006)*

This also holds challenges for Singapore’s veterinarians, as most veterinarians are trained in temperate regions, and may not have had exposure to diseases that come with tropical aquaculture.

#### 4.8.3 Making someone a model case

When asked about what potential interventions could reduce antibiotic use, most of the participants agreed that there had to be a “model farm” operating successfully within the three themes before they consider implementing any intervention rolled out by AVA:

“*I have to be a shining example of success then I can tell them I am very successful [even without antibiotics]*. *If I cannot show them that I am so successful*, *I say no use also… I mean AVA can appeal but if you are struggling to make ends meet then it’s very hard to say oh… please do this please do that*, *you are required to do that” (ID001)*

### 4.9 Relationship with AVA and peers

We also explored the relationship dynamics between participants and their peers as well as with regulatory bodies (i.e. AVA). In terms of relationships with other fish farmers, most participants said that they enjoyed cordial interactions and professional relationships with their counterparts. These relationships proved beneficial in some situations, such as when they need to come together to buy feed in bulk at a lower price. Although participants felt comfortable sharing their experiences about fish fry suppliers or quality of feed with other fish farmers, participants said that they generally refrained from giving formal advice to their counterparts for a few reasons. Participants also believed that each fish farm was unique–what worked for theirs may not work for another–and they did not want to shoulder the blame if anything went wrong. Additionally, they believed that trial and error with one’s own farming practice was a rite of passage of becoming a fish farmer, and the most effective way of learning:

*“Yeah*, *of course, when you have up to a certain amount of years of experience, [you’ll] naturally know what to do, you know? It becomes like a very natural kind of instinct…and when the fish need help or don’t need help, you’ll know” (ID009)*

In terms of AVA’s involvement in their faming practice, we found that participants had mixed responses about AVA’s involvement in their farming practice. For instance, some participants found AVA’s advice about fish farming practices generally helpful, and they were able to implement suggested best practices. However, a few participants expressed frustration with restrictive regulations in the absence of alternatives or other guidelines for treatment:

*“They only give us the regulation*, *you cannot do this, cannot do that, what is the other option, no other option, then when the farmer see the fish dying, they start thinking what should I do, then no other method, then only one thing they can think of antibiotics right, they give them fish, antibiotics, they say okay then you too much, what is not too much, how much is a measurement, what is not too much, there is no guidelines, don’t give too much, how? (ID005)*

## 5. Discussion

To our knowledge, this study is the first qualitative investigation of the factors that work together to influence fish farmers’ decisions to use antibiotics as therapeutic treatments in their farming practices. Our findings indicate that fish farmers in Singapore generally do not use antibiotics sub-therapeutically in their practice. Farmers who use antibiotics in their farming practice generally use them as a last resort or for specific conditions.

Our findings indicate that farmers’ use of antibiotics was influenced mainly by three factors: their personal experience and knowledge, the regulatory environments in which they rear and sell fish, the business aspect, as well as public perception and consumer awareness. At the individual level, personal knowledge and experiences about antibiotics gained through individual farming experimentation influenced farmers' decisions about antibiotic use. Some examples given by farmers included noticeable impaired growth of fish post antibiotic consumption and the futility of feeding antibiotics to sick fish because of their lack of appetite. In place of antibiotics, participants generally found better alternative strategies to increase fish survival, including purchase of better quality fish fry, good hygiene practices, as well as other non-conventional forms of disease management. This was consistent with previous research about increasing fish survival [11, 2].

In terms of regulatory environments, we found that regular oversight from AVA as well as the stringent antibiotic-free requirements by fish harvesters and exporters played an important role in deterring antibiotic overuse. Participants also discussed how the regulatory environment has led to some noticeable behavioural changes as fish farmers become more aware about chemicals they use in their farming practice. At the economic level, the relatively high price of antibiotics in Singapore coupled with stiff competition from neighbouring countries was a strong disincentive for participants to use antibiotics in their farming practice. This was particularly salient among farmers raising lower-value food fish such as tilapia and milk fish. Lastly, we found that fish farmers were sensitive to increasing consumer awareness about food safety, and participants were wary about feeding fish antibiotics as it may not be acceptable to consumers.

We also found that some of these factors, particularly cost and consumer acceptance, did not only apply solely to antibiotic interventions; farmers had similar concerns with other interventions such as fish vaccination and veterinary prescription. Additionally, they raised some concerns about the feasibility of implementing these interventions. Consequently, participants offered that in order for intervention uptake, there had to be a “model farm” to show the practicality and success of the intervention navigating within the three parameters. Further, participants had mixed responses about AVA’s role in their farming practice. While some participants discussed the benefits of their involvement, others shared the challenges of any implemented regulation without an alternate option, and the challenges of running a profitable business within a restrictive environment.

Since our study only focused on small-scale food fish farmers in Singapore, we did not have any data on antibiotic use in larger commercial operations, where antibiotic use may be driven by more intensive production systems and export requirements. Limited resources also meant that we did not explore the substantial ornamental fish industry locally, where higher value fish might provide more incentives for antibiotic use. In addition, as local small-scale food fish farmers are a challenging population to reach out to, we recruited most of our farmers at a workshop in AVA and this may have introduced some self-selection bias, as not all fish farmers attend these workshops. Due to the self-reporting nature of our study, it is possible that participants may have provided inaccurate information about their antibiotic use. However, our qualitative approach allowed for an in-depth analysis of factors influencing behaviour, which is challenging to elicit from surveys.

## 6. Policy implications

Our analysis showed farmers’ use of antibiotics is often a rational behaviour that takes into account personal, regulatory and market factors, and this has key implications for future policy established for small-scale aquaculture businesses. For instance, any proposed policies to reduce the need for antibiotic treatments need to take into account the high operation costs, low product value and stiff external competition from neighbouring countries, especially for small-scale food fish farmers who make up the majority of the fish farmer population in Singapore. Similarly, positive behaviour change is more likely if reinforced with economically viable role model fish farms. Additionally, in the absence of feasible alternatives or standard treatment guidelines, it remains futile to frame changes in antibiotic use behaviour negatively. Reduced consumer acceptance of products extensively treated with antibiotics is also likely to have a major influence on fish farmers’ use of antibiotics, suggesting that policies relating to public education on antibiotics and resistance should also include material on the use of antibiotics in food production.

## 7. Conclusion

Although food fish farming industries differ significantly across countries, lessons learnt from the preliminary results in Singapore’s food fish farming industry demonstrate the importance of an environment in which multi-dimensional factors come together to discourage the irrational use of antibiotics in food animal production. In addition, our results allow greater insight into food fish farmers’ perspectives on infection control and form a basis from which further research work can be undertaken. Some examples include interventions to increase farm biosecurity, further communication about clearer standard treatment guidelines as well as awareness about the benefits of food fish vaccination.

## Supporting information

S1 Appendix (DOCX)
