# Supplementary material for: Determinants influencing antibiotic use in Singapore's small-scale aquaculture sectors: A qualitative study
Source: PLoS One. 2020 Feb 25;15(2):e0228701. doi: 10.1371/journal.pone.0228701 (PMC7041790; doi:10.1371/journal.pone.0228701)
Supplement: S1 Appendix — (DOCX) [file pone.0228701.s001.docx]

Appendix A

**Knowledge, attitudes and practices surrounding the use of animal antibiotics in Singapore**

*Farmers in-depth interview guide*

*Farming business: type of farm, animal health, experiences with animal diseases and farming business*

1. Can you tell us about your farm? [Prompt for description about farm size, herd size, number of works, farm environment and technology]
2. How many chickens/pigs/fish do you usually keep in your farm?
3. Has your farm been affected by an outbreak of disease in the past? How did this impact the health of your animal and your business? What is the current health state of your animals?
4. Do you have any measures in place to prevent and control animal diseases?
5. Whom do you sell your chickens/pigs/fish to?
6. How well are you doing in your farming business? Do you experience any difficulties in running your farming business? If yes, can you share with us what they are?

*Antibiotics use:*

1. Do you use antibiotics for your farm? [Probe by giving names of some common antibiotics used in animal farming]
2. When do you use antibiotics for your animals? [Prompt for frequency and purpose]

[If for prophylaxis or growth promoters]:

- 1. How frequent do you give your animals antibiotics?
  2. Do you give antibiotics during certain growth periods (breeding, nursery, fattening for pigs) or the entire time when the animals are in the farm?
  3. How do you administer the antibiotics?
  4. How much antibiotics do you give each time?

[If for disease treatment]:

- 1. Which signs of animals will prompt you to use antibiotics?
  2. When will you stop giving animals antibiotics?

1. What kinds of antibiotics do you often use?
2. Who makes the decision to use antibiotics in your farm?
3. How do you decide which kind of antibiotics to use and at which dose?
4. Where do you get information about antibiotics from?
5. Who do you talk to when you have difficulty in running your business, managing your farm or control the infections in your herds?
6. How important do you think are antibiotics in running your farm?

*Suppliers of antibiotics*

1. Where or from whom do you get your antibiotics?
2. How do you learn about this place/person?
3. How long have you been getting antibiotics from them?

*Antibiotic resistance:*

1. Have you heard of the term antibiotic resistance? If yes, what does it mean to you?
2. What do you think are the positive and negative impacts of antibiotic usage in your farm?
3. Do you think if there is sufficient information and guideline about how to use antibiotics to treat animals?
4. How confident you are that you are using the adequate amount, not too little and not too much, of antibiotics?
5. How confident you are that your antibiotics are effective in managing illnesses in your fish?
